# Enhanced residual-attention deep neural network for disease classification in maize leaf images

**DOI:** 10.1038/s41598-025-14726-1

**Published:** 2025-08-12

**Authors:** Nidhi Parashar, Prashant Johri, Ahmed Elbeltagi, Ali Salem, Prakash Choudhary, Vijay Kumar, Tarun Agrawal

**Affiliations:** 1 School of Computer Science and Engineering , Galgotias University, Noida, Uttar Pradesh 201308 India; 2 School of Computer Application and Technology, Galgotias University, Noida, Uttar Pradesh 201308 India; 3 Agricultural Engineering Department, Faculty of Agriculture, Mansoura University, Mansoura, 35516 Egypt; 4 Civil Engineering Department, Faculty of Engineering, Minia University, Minia, 61111 Egypt; 5 Structural Diagnostics and Analysis Research Group, Faculty of Engineering and Information Technology, University of Pécs, Pécs, 7622 Hungary; 6 Department of Computer Science & Engineering, Central University of Rajasthan, Rajasthan, India; 7 Department of Information Technology, Dr. B.R. Ambedkar National Institute of Technology, Punjab, India; 8 Department of Computer Science Engineering & IT, Jaypee Institute of Information Technology, Noida, Uttar Pradesh 201309 India

**Keywords:** *Maize disease* classification, *Attention mechanism*, *Residual learning*, *Deep learning*, Plant sciences, Biomedical engineering

## Abstract

Disease classification in maize plant is necessary for immediate treatment to enhance agricultural production and assure global food sustainability. Recent advancements in deep learning, specifically convolutional neural networks, have shown outstanding potential for image classification. This study presents Maize Net, a convolutional neural network model that precisely identifies diseases in maize leaves. Maize Net uses an attention mechanism to increase the model’s efficiency by focusing on the relevant features and residual learning to improve the gradient flow. This also addresses the vanishing gradient problem while training deeper neural networks. A five-fold cross-validation test is conducted for generalization across the dataset, generating five models based on distinct training and testing sets. The macro-average of all evaluation metrics is considered to address the dataset’s class imbalance problem. Maize Net achieved an average F1-score of 0.9509, recall of 0.9497, precision of 0.9525, and classification accuracy of 0.9595. These outcomes demonstrate MaizeNet’s robustness and reliability in automated plant disease classification.

## Introduction

Maize is a flexible crop in terms of variety and usage. It is grown in tropical, subtropical, and arid regions. It is the second-most widely grown crop in the world. With a global average yield of more than 5 tonnes/ha, more than 170 nations are producing approximately 1147.7 MM tonnes of maize on a total area of 193.7 MM. Global consumption of maize primarily involves feeding (61%), foodstuffs (17%), and the manufacturing industry (22%). With 83% of worldwide production going towards animal feed, starch, and biofuels, it has become a major commercial crop ICAR, 2024^1^. According to DACNET, 2020^2^, India grew maize over 9.2 million hectares in 2018–19. In 2021, maize produced the highest amount at 1.2 billion tons, with the fastest growth rate at 104% since 2000 FAOSTAT, 2021^3^. APEDA, 2024^4^ reported that India produced approximately 35.91 million tonnes of maize in 2022–2023 contributing significantly to the global maize production.

Maize’s vulnerability to diseases might result in substantial crop losses. Some common diseases affecting maize plants include the Streak Virus, transmitted by maize-carrying leafhoppers; Northern Leaf Blight, triggered by the fungus Exserohilum turcicum; Southern Leaf Blight, instigated by Cochliobolus heterostrophus; Grey Leaf Spot caused by the fungus Cercospora zeae-maydis; Common Rust caused by the fungus Puccinia sorghi; Maize Dwarf Mosaic Virus transmitted by aphids; Corn Smut caused by the fungus Ustilago; Fusarium Ear Rot caused by various Fusarium species; and Bacterial Stalk Rot caused by bacterial pathogens, such as Erwinia chrysanthemi, which can result in yield loss. Maize is a globally traded commodity. Diseases that affect maize crops may have implications for international trade and food supply chains. Ensuring the health of maize crops through disease classification is essential for maintaining stable trade relationships and meeting global food demand.

Manual disease recognition in maize is time-consuming because it requires a physical examination of the plants to look for signs of deformity, discoloration, lesions, or odd growth patterns. Machine learning (ML) models learn, identify patterns, and detect the disease with no human intervention. Ideally, machines are supposed to enhance accuracy and effectiveness while lowering the possibility of errors made by humans, as stated in Zhong et al.^5^. Deep learning has been widely used in disease classification across a wide disease spectrum^6,7^. Al-Jebmi et al.^8^ proposed a two-component deep neural network with a dense-branched block and a feature-synthesis block boosted by the Gaussian process, to recognize small lesions in images. LeCun et al.^9^ have widely employed deep CNNs for image-based predictive modelling due to their effectiveness. In recent years, many researchers have attempted to detect plant diseases using CNN because of their capability of learning and extracting features from the images on their own. Priyadharshini et al.^10^ changed the LeNet and developed a deep CNN model to classify diseases damaging maize leaves. The test was performed on the maize sample of the plant village dataset used in Mohanty et al.^11^, and the CNN was trained to differentiate between four leaf classes. The model’s accuracy was 97.89%.

Feature extraction is necessary for disease classification tasks due to the various morphological patterns observed on leaves. This process allows for the extraction of the most pertinent and distinguishing features from plant leaf images, resulting in improved classification performance. A CNN architecture typically includes a feature extraction module for image classification problems, followed by a classification module. The feature extraction module usually consists of convolutional layers, activation functions to introduce non-linear transformations, and pooling layers for feature map-down sampling. Amer et al.^12^ proposed a VGG19 model to extract initial features, followed by a vision transformer for extracting deep features. The hybrid feature extraction approach enhanced the classification accuracy of the classification model significantly. Stefania et al.^13^ extracted texture-based features from RGB plant leaf images and then used a support vector machine with an RBF kernel for image classification. Roy et al.^14^ effectively employed attention mechanisms in the feature extraction module to enhance the deep learning model’s classification performance through feature recalibration, Zeng et al.^15^ developed a self-attention CNN that diagnoses diseases with 95.3% and 98% accuracy on two datasets, respectively. Chen et al.^16^used pre-trained Mobile Net with a squeeze-and-excitation block. Chen et al.^17^developed the DenseNet-based Mobile-DANet model. They used depth-wise separable convolutions in dense blocks and then an attention module to find the feature inputs’ inter-channel correlation. CNN models with attention-based features improve their performance in classifying plant diseases.

In addition to an efficient feature extraction process, another challenge with very deep CNNs is that they suffer from vanishing gradients during model training, in which the gradients become very small while propagating backward through multiple layers. It hampered the model’s learning process. ResNet was proposed in He et al.^18^ addresses this issue by providing skip connections. Hassan et al.^19^ built a novel CNN using inception network and residual mapping for enhanced feature extraction and used depth-wise-separable convolution operation for minimizing parameter generation. Dhruvil et al.^20^ proposed a residual student/teacher architecture based on the Xception model. The model employs residual blocks in encoder-decoder blocks for addressing exploding or vanishing gradients. Zhao et al.^21^ proposed an attention-based CNN with inception blocks and residual modules and achieved 99.55% classification accuracy. Although several models have demonstrated satisfactory performance, their interpretability and generalization abilities have not been investigated. It is imperative to create techniques that are effective for a range of plant diseases.

This paper aims to design a deep CNN model for maize plant disease classification using a small dataset. The following are the proposed study’s main contributions:

- MaizeNet employs spatial and channel-wise squeeze-excite network and residual learning to enhance feature extraction and training stability.
- The performance of the proposed model MaizeNet, is evaluated on a small maize dataset having four classes of maize leaves using 5-fold cross-validation for model generalization across the dataset.
- To provide a fair assessment of the model’s performance on an unbalanced dataset across all classes, the macro average is used to evaluate the proposed MaizeNet model. The results show that MaizeNet can accurately classify the maize plant leaves.
- To the best of our knowledge, no study has proposed an identical framework for identifying maize plant diseases.

The remaining part of this research study is organized as follows: Sect. 2 presents the relevant work; Sect. 3 covers the details of the maize dataset and the proposed MaizeNet design. Section 4 includes the evaluation measures and experimentation settings. Section 5 covers results, discussion, and comparison with existing models for maize disease classification. The research concludes in Sect. 6.

## Related work

This section examines the related research on plant disease classification to offer background and perspectives.

Chen et al.^22^ retained the initial pre-trained layers of the VGG19 model and replaced the end layers with a convolutional block, which consists of a convolution layer followed by a batch normalization layer and Swish activation. This convolutional block is followed by a pair of Inception blocks. In Sibiya et al.^23^, VGG16 model was used to identify common rust infection in the maize leaves at the premature, middle, and final phases. They set up Otsu’s thresholds for image segmentation and adopted Fuzzy decision criteria for obtaining features. The model obtained 95.63% accuracy on the validation set and 89% accuracy on the testing set. Waheed et al.^24^ proposed an optimized DenseNet121 for maize disease classification. They collected 12,332 images from multiple places with a 250 × 250 resolution. They trained four popular CNN models, namely, VGG, EfficientNet, XceptionNet, and NASNet, and compared the optimized model with them. The proposed optimized model classified maize leaf images with 98.06% accuracy.

Haque et al.^25^ implemented three designs on the Inception-v3 model: the first included a flattened layer with a fully connected (FC) layer, the second had a global average pooling (GAP) layer, and the third had a GAP layer with a fully connected (FC) layer. Amin et al.^15^ A maize plant disease classification framework was proposed using EfficientNetB0 and DenseNet121.The concatenating approach merges the features extracted by each CNN, producing a more intricate feature set. The proposed approach could categorize data with 98.5% accuracy. Subramanian et al.^26^ used pre-trained VGG16, InceptionV3, ResNet50, and Xception models for recognizing different kinds of maize leaf diseases. Hyperparameter values are optimized using Bayesian optimization. All four pre-trained models classified maize leaf diseases with more than 93% accuracy. The model proposed by Xu et al.^27^ employed a multiscale CNN to enhance the accuracy of identifying maize diseases. They modified AlexNet proposed in Krizhevsky et al.^28^ by adding a convolutional layer and an Inception component in the feature extraction block. Then, they used a FC layer with the GAP layer to prevent over-fitting due to excessive parameters.

Owing to the large number of parameters, pre-trained models employed in the transfer learning techniques are vulnerable to overfitting problems on small plant disease datasets. Developing a CNN from scratch offers flexibility in handling distinct disease patterns. Sun et al.^29^ developed a framework based on CNN to identify maize disease. The improved retinex model proposed by Shen et al.^30^ handled the data sets and addressed the issue of improper classification caused by bright light, isolating brightness and reflection components to improve the perception of colors. The unhealthy leaf anchor container was adjusted using the enhanced region proposal network used by Yu et al.^31,32^ and Haggag et al.^26^. The communication module transforms the feature map of the fine-tuning module into the detector module and combines the features to increase the recognition precision of diseases. Sibiya et al.^33^ developed a GUI-based CNN for recognizing and categorizing diseases affecting maize plants. Disease classification using Neuroph Studio empowered the CNN design with feature extraction capabilities present in the package library.

A deep CNN model called NPNet-19 was proposed by Nagaraju et al.^34^ to detect crop diseases in maize. They used maize leaf images from the publicly available PlantVillage dataset and a Kaggle dataset to train the model. Their model exhibited 97.51% accuracy on training datasets and 88.72% accuracy on test datasets. Haque et al.^35^ employs images from the Plant Village collection to propose a new CNN technique for maize crop disease classification. To identify the unseen maize crop images, the proposed approach exceeded the top-performing DenseNet121 by 3.2% in predictions. Hari et al.^36^ trained a CNN model using eight categories of plant leaves. The model incorporated one million parameters and resulted in a 99.14% accuracy. Thakur et al.^37^ proposed employing a CNN enabled with Vision Transformer for identifying diseases in maize leaves. The proposed approach successfully recognizes multiple plant diseases for various crops by combining the capabilities of Vision Transformers with a standard CNN. The accuracy for identifying plant diseases reached 92.59% on maize datasets. Theerthagiri et al.^38^ proposed the pre-trained VGG-16, ResNet-34, ResNet-50 models for the maize disease classification on PlantVillage dataset. In this study, ten-fold cross-validation was used for the generalization of the model. Thakur et al.^39^ proposed the ConViTX architecture that integrates convolutional neural networks with vision transformers to concurrently record local and global data. ConViTX model is evaluated on four different datasets and outperformed state-of-the-art models.

Existing attention-based and residual CNN models have demonstrated success in general image classification tasks, yet they exhibit key limitations when applied to domain-specific problems. These models are often designed for large-scale, balanced datasets and do not account for the fine-grained visual features—such as localized discoloration, vein degradation, or fungal textures—characteristic of diseased maize leaves. Furthermore, conventional architectures may struggle to generalize effectively on small agricultural datasets due to overfitting or insufficient feature sensitivity. To address these gaps, we propose MaizeNet, a lightweight CNN model that incorporates spatial and channel-wise squeeze-and-excitation mechanisms with residual learning. This architecture enhances the network’s ability to focus on biologically relevant features while maintaining training stability, even under data scarcity and class imbalance. By explicitly tailoring the model to the challenges of agricultural image analysis, MaizeNet offers a more effective and domain-appropriate solution compared to existing approaches. Table 1 presents a summary of relevant studies focused on maize plant disease classification.

**Table 1:** A summarized view of relevant studies focused on maize disease classification.

Research Papers	Model	Pre-processing	Augmentation	Remark
[15]	Attention-CNN	resizing to 168 × 168	No	The model has 3 residual modules, 3 convolutions, and a GAP layer, followed by a SoftMax.
[17]	Attention-based DenseNet	Filtering, resizing to 224 × 224, edge filling, sharpening	Yes	Usage of depth-separable convolutions in dense blocks along with the attention mechanism
[22]	VGG + Inception	Filtering, resizing to 224 × 224, sharpening	Yes	The final convolutional layers of VGG were replaced with a convolution layer, batch normalization, and Swish activation.
[23]	VGG16	-	No	Using Otsu threshold segmentation, they differentiated the images into two categories: pixels with bright intensity and pixels with darker intensity.
[24]	Modified DenseNet	-	Yes	A dense block layer contains the Batch normalization layer, ReLU activation, conv(3 × 3), and Dropout. The layer amid two dense blocks performs downsampling.
[25]	Modified Inception-v3	resizing to 256 × 256	Yes	Three different Inception-v3-based models are developed
[20]	EffcientNetB0 + DenseNet121	resizing to 244 × 244	Yes	Merged features from multiple pre-trained CNNs using a concatenation technique.
[26]	VGG16, InceptionV3, ResNet50, Xception	resized to 224 × 224 , 299 × 299, 96 × 96 for different models	Yes	Tuned hyperparameters using Bayesian Optimization
[27]	TCI-AlexN	-	Yes	The model improves AlexNet including a 3 × 3 × 256 convolution after the last layer for pooling.
[29]	CNN trained from scratch	Cropping, expanding, mirroring	Yes	The potential areas for recognition are removed by sharing the features of the transmission module layer by layer.
[33]	CNN trained from scratch	-	No	Used image feature of Neuroph studio for model training
[34]	CNN trained from scratch	resizing to 224 × 224, rescaling	Yes	The finest model featured an image of 224 × 224 size, a batch of 32 samples, a 3 × 3 kernel size, and a train-test split of 80:20.
[35]	CNN trained from scratch	resizing to 227 × 227, rescaling	Yes	With three times fewer parameters to train, the model showed a 3.2% rise in prediction accuracy compared to the top-performing pre-trained network.
[36]	CNN trained from scratch	resizing to 224 × 224	Yes	The model has three layers of convolution, each linked to a pooling layer followed by the matching dense layers.
[37]	Plant-Xvit	-	No	The Conv2D blocks of the VGG and Inception model, with the ViT elements MLP and MHA with linear estimates, are the principal components.

## Materials and methodology

The present section explains the maize dataset and the MaizeNet model proposed in the research. Section 3.1 explains the data and data pre-processing steps. Section 3.2 covers the proposed MaizeNet model architecture in detail.

### Dataset and its pre-processing

To collect the maize dataset, we searched the Kaggle and GitHub data sources. The dataset prepared by Ghose et al.^40^ it is a public dataset available on the Kaggle website. It consists of a total of 4188 leaf image samples with 1146 samples of leaves infected with blight, 1306 samples of leaves infected with common rust, 574 samples of leaves infected with gray leaf spot, and 1162 samples of healthy leaves with 256 × 256-pixel resolution.

The maize dataset^40^ is curated from the PlantVillage dataset used in Geetharamani et al.^41^ and the PlantDoc dataset introduced by Singh et al.^42^ combining the maize leaf images based on disease classes. The PlantVillage dataset has 61,486 plant images from 39 different kinds of plants. The plant images are captured in lab settings and not in actual conditions of agricultural fields, so their practical utility may be limited. The PlantDoc dataset, which was used to develop the maize dataset, is also a public dataset that includes real-world images of healthy and diseased plants. It consists of 2,598 plant images covering 13 kinds of plants and 27 distinct categories.

Table 2 presents the details of the maize dataset, which is used in the current research. Of the total 4,188 images, 3,838 images (approx. 91.6%) originate from the PlantVillage dataset, which includes lab-captured images under controlled lighting and background conditions. The remaining 350 images (approx. 8.4%) are from the PlantDoc dataset, which contains real-field images captured under natural conditions. This combination allows for both controlled evaluation and preliminary validation under real-world scenarios.

**Fig. 1:**
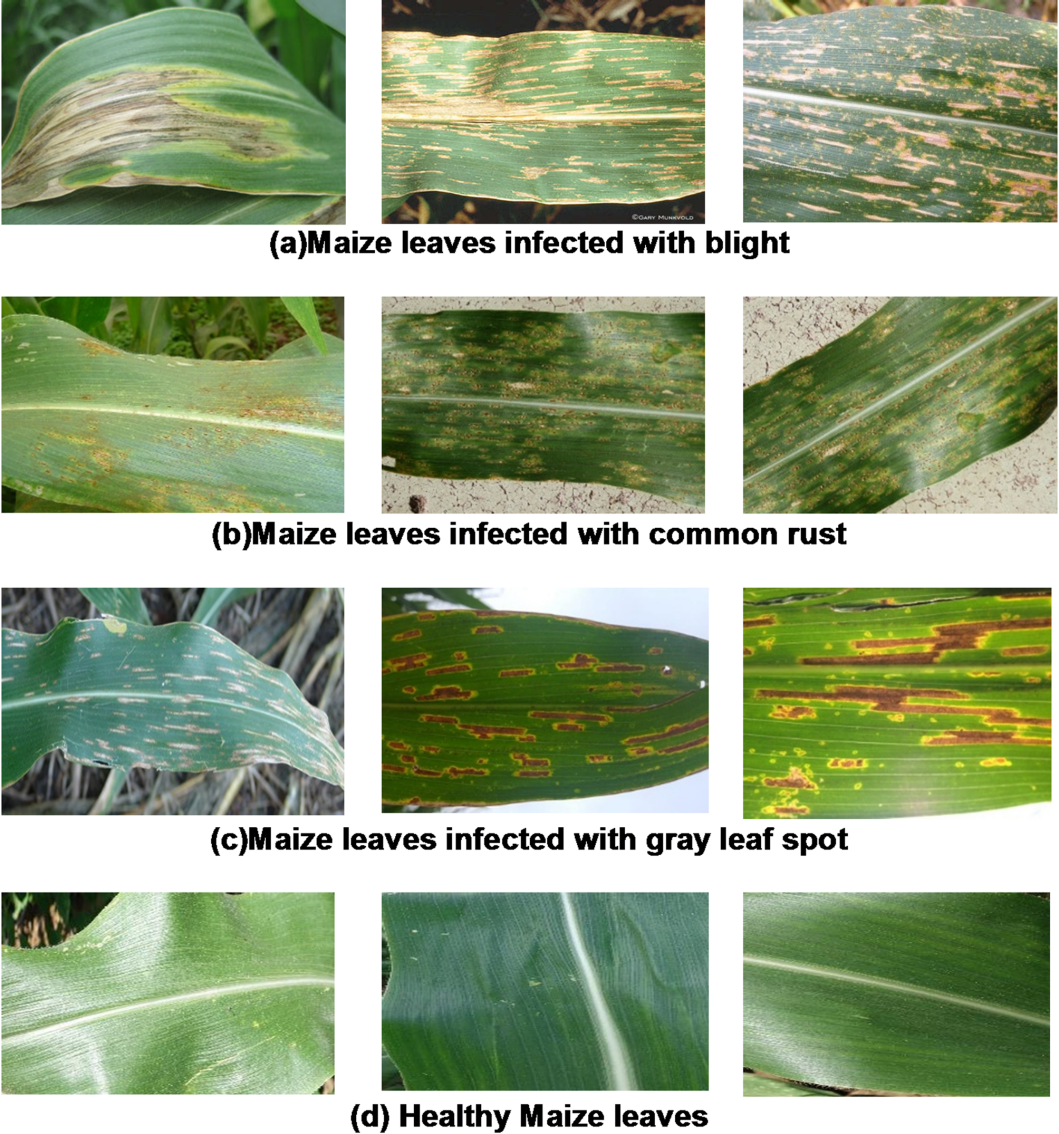
Maize plant leaves infected with blight, infected with rust, infected with gray leaf spot and healthy leaves

In the dataset pre-processing, the images are initially resized to a standardized dimension of 224 × 224 × 3 to ensure uniformity for integration with neural networks and increase computing efficiency throughout model training. The pixel values are divided by 255 to keep normalized values within the [0–1] range to enhance model convergence and stability during model training and optimize gradient descent. Considering the dataset’s small size, data augmentation was used in Han et al.^43^ helps to improve the model’s generalization ability by increasing the number of images for model training. The operations such as rotation (0° to 20°), horizontal flipping, zooming, and shear transformation with a range of 0.2 generate an augmented dataset. The augmentation is exclusively applied to the training data to prevent overfitting, and the ImageDataGenerator function from Keras is utilized for real-time data augmentation, contributing to model robustness. Leaf images from the obtained maize dataset are presented in Fig. 1.

**Table 2:** Description of the maize dataset.

Class	Instances	Original Resolution	Input Resolution
Common Rust	1306	256 × 256	224 × 224
Blight	1146	256 × 256	224 × 224
Healthy	1162	256 × 256	224 × 224
Gray Leaf Spot	574	256 × 256	224 × 224
Total Images	4188	---	---

### Prediction models

This paper presents a MaizeNet model, relying on the growing popularity of deep learning, especially CNN, in image classification. Encouraged by the achievements of previous studies, our approach explains the specific structure concepts and training processes used in the present study to improve the effectiveness of the proposed approach for plant disease classification. The systematic approach employed to develop our existing understanding and use of CNNs in this critical field is explained thoroughly in the subsequent subsections.

#### Problem formulation

The concept of supervised learning is applied in the present study to categorize diseases observed in the maize plants. Let us postulate that there are $\:{T}_{i}$ training samples in the maize leaf dataset, $\:\:=\:\{\text{A},\:B\}$ A denotes the input leaf images, and B is a representation of their original labels. We depict the training samples as, $\:A={\{a}_{1}{,a}_{2},\dots\:..{a}_{Ti}\}$, and the original label linked to every sample as $\:B={\{b}_{1}{,b}_{2},\dots\:..{b}_{Ti}\}$. The $\:{B}_{i}=\in\:\:[1,\:2,\:\text{3,4}]$, where 1,2,3, and 4 represent the common rust, blight, healthy, and gray leaf spot leaf classes, respectively. The classifier’s output, denoted as $\:{f\:}^{\left(w\right)}:\:A\to\:Z$ where (w) represents the parameters, may deviate from the true label B. This variation, also known as the prediction error rate, represents the variance between the true label $\:\left(B\right)$ and the estimated label $\:\left(Z\right)$. The parameters are repeatedly updated to minimize this error rate throughout training.

#### MaizeNet architecture

Increasing the depth of deep learning architectures may result in higher training error and accuracy. As depth increases, accuracy may become saturated, leading to a rapid rise in model loss, an event called as degradation. The vanishing gradient problem, in addition to degradation, presents further challenge in deep learning models. He et al.^18^ implemented residual learning to overcome these challenges. He et al.^18^ additionally applied identity mapping to enhance the generalisation of the deep model architecture. The architecture proposed in this study has five attention based-residual blocks. Increasing the depth further can increase the training parameters which can lead to overfitting and degradation as discussed above. Furthermore, increase in training parameter can also increase the high computational complexity.

In the MaizeNet there is an initial block followed by four attention-based residual blocks. The initial block has the convolutional, batch normalization, and attention (LeakyRelu) layer. Meanwhile, each attention-based residual blocks have a convolutional block, a channel spatial squeeze-excite (csSE) block^14^ for the attention mechanism, and a strided residual layer. In every convolutional block, there is one convolutional layer, which is followed by a batch normalization layer and ReLU activation. An attention mechanism incorporated into the architecture allows intermediate feature maps to be recalibrated. To achieve this, csSE block has been used in the entire architecture. Traditionally, SE blocks^44^ identify significant features, however the csSE attention mechanism not only determines which features are important but also locates their spatial location.

Additionally, the architecture also comprises the strided residual layer, which uses the strided convolution which enabling better feature extraction. Instead of employing average or max pooling, strides are used in the proposed architecture to perform downsampling. It regulates the rate at which the convolutional filter scans the input, resulting in lower spatial resolution of the feature maps and achieving downsampling. Using of strided convolution helps in downsampling as well as in feature extraction, while in pooling there is only the downsampling. Other than these five attention-based residual blocks, MaizeNet has one GAP layer followed by three FC layers, and two dropout layers. To the author’s best knowledge, no other study has proposed a similar architecture for the four-class maize disease classification.

**Fig. 2:**
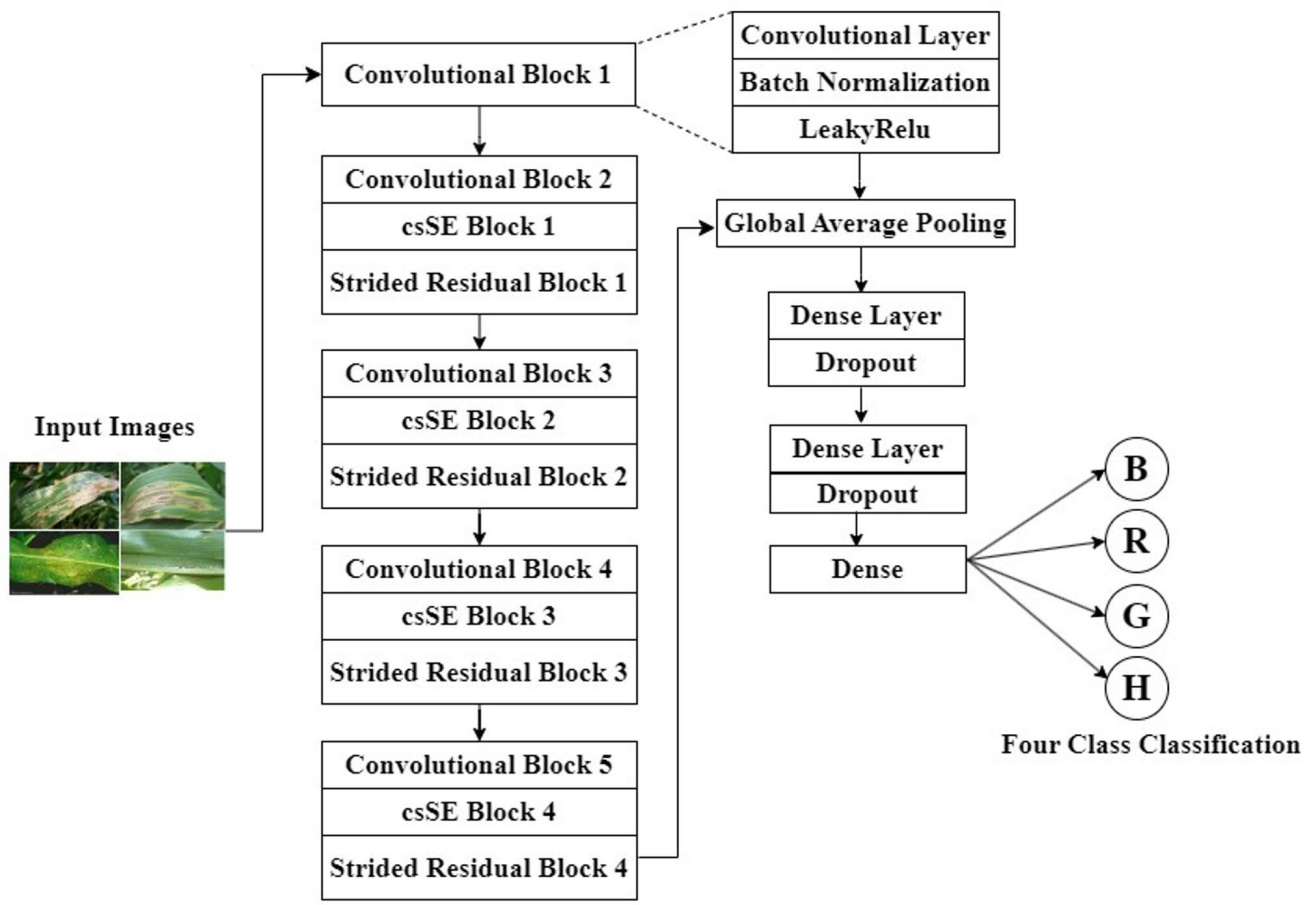
The block diagram of the MaizeNet Model

Figure 2 shows the block diagram of the MaizeNet model. Table 3 presents a layer-by-layer comprehensive outline of the proposed MaizeNet model. The model comprises approximately 2.1 million parameters to train during the model-training process. A description of the various architectural components and layers is provided below.

### Initial block

The initial block in the architecture takes the input images having a 224 × 224 × 3 resolution. This block comprises three layers. The starting layer is the convolutional layer having 16, 3 × 3 filters. By convolving over input data, these learnable filters enable the network to identify and extract hierarchical features. The layer adds the necessary padding to the input to ensure its identical spatial dimensions as the output feature map and retains the spatial information during the convolution operation. During the convolution process, filters $\:\left(W\right)$ convolve with the input $\:\left(X\right)$ and generate the activation maps$\:\left(Y\left[:,:,i\right]\right)$. These activation maps are stacked along the depth, and a 3D tensor $\:\left(Y\right)$ with dimensions $\:({P}^{{\prime\:}},{Q}^{{\prime\:}},K)$is produced as the output. Equation (1) defines this process mathematically.

1 $$Y[:,:,i] = \sum\limits_{p,q,c} W [:,:,c,i] \cdot X[p,q,c]$$

Here, p and q represent the spatial dimensions of input along the vertical and horizontal axes, and c represents the number of filters. A batch normalization follows this conv2D layer to normalise the input distributions, reduce inside co-variate shifts, and enable the increased learning rates during the training process. A LeakyRelu activation layer follows the batch normalization layer to include non-linearity, which is required for the model’s ability to learn and represent complex input-to-output mappings. L2 regularisation with a coefficient of 1e-4 is used to avoid overfitting and to promote steady learning. The kernel is initialised using the ‘He normal’ method.

### Channel Spatial squeeze excite (csSE) block

**Fig. 3:**
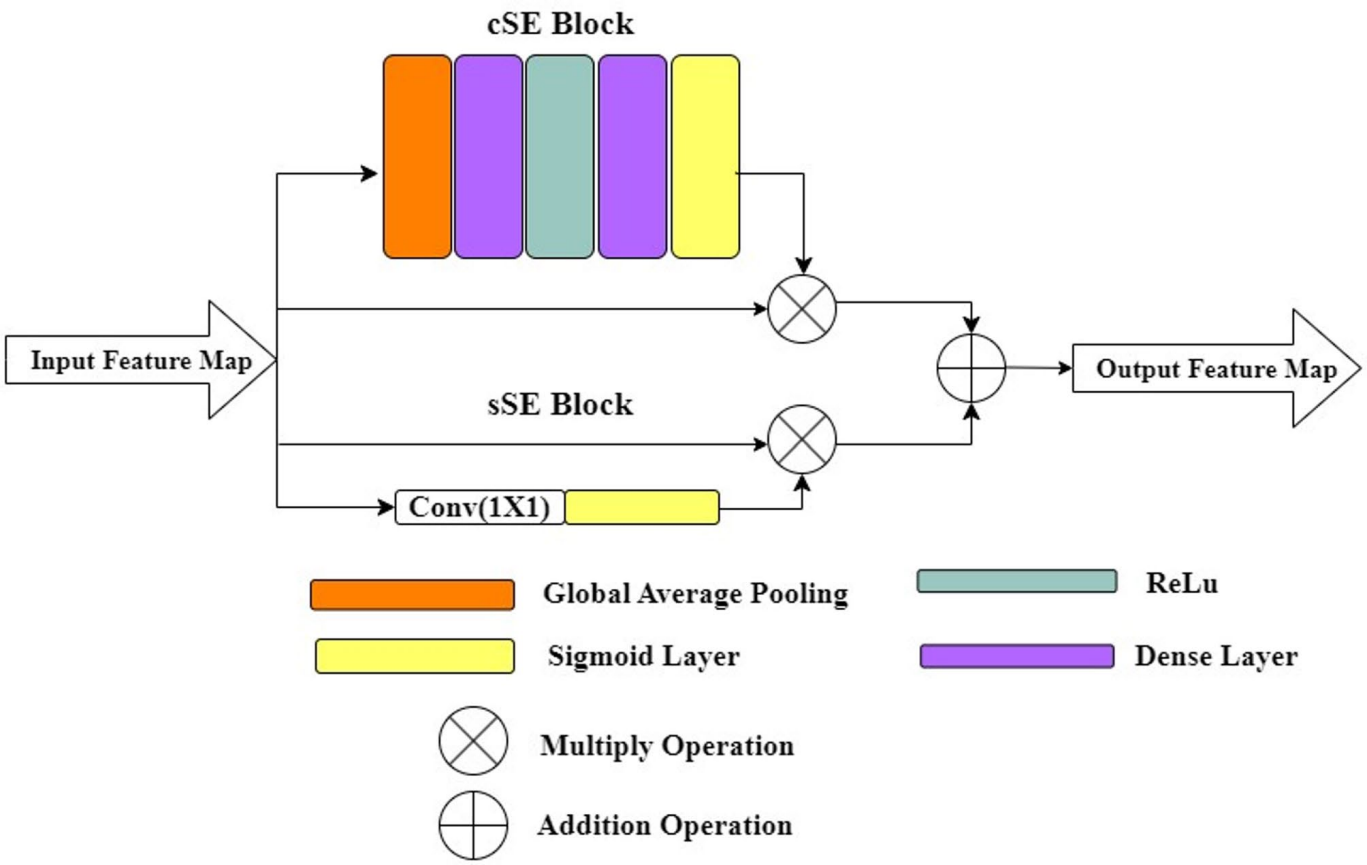
The block structure of channel spatial squeeze-excitation(csSE) module^14^

Attention techniques enhance feature extraction which results in enhance model performance on tasks like identifying objects and classification by focusing on the significant regions in an input image. It offers enhanced control over interaction between objects and better spatial resolution. Hu et al.^44^ introduced the Squeeze and Excite network (SE), which improved network performance by explicitly modelling interrelationships between channels through channel-wise feature response calibration and enabling the network to adapt to the relative significance of various channels in the feature maps. Roy et al.^14^ proposed a csSE block by combining a newly developed spatial SE (sSE) block with the channel-SE block (cSE)^44^. The cSE block modifies an input tensor by first employing global average pooling and then using two dense layers with ReLU and sigmoid activations to generate attention weights. The final output is obtained by element-by-element multiplication of the original input and the attention weights. To enhance feature representation, this method dynamically modifies channel significance. sSE utilizes a (1 × 1) convolutional layer to generate spatial attention weights from the input tensor. It then multiplies the input tensor by these weights, enhancing spatial information. csSE block combines both channel-wise(cSE) and spatial (sSE) attention mechanisms. The final output is obtained by adding channel-wise and spatial attention-enhanced tensors element-by-element. Figure 3 presents the architecture of csSE block.

### Strided residual block

There are four strided residual blocks in the proposed architecture for extracting the features. Residual connections skip one or more neural network layers and add the input of a layer to its output, enabling residual functions to be learned by the network. Such connections make it easier to train extremely deep neural networks by alleviating the vanishing gradient problem and enabling smoother optimization. Strided residual blocks use strided convolutions in residual connections for improved feature extraction and an efficient feature map reduction process.

**Fig. 4:**
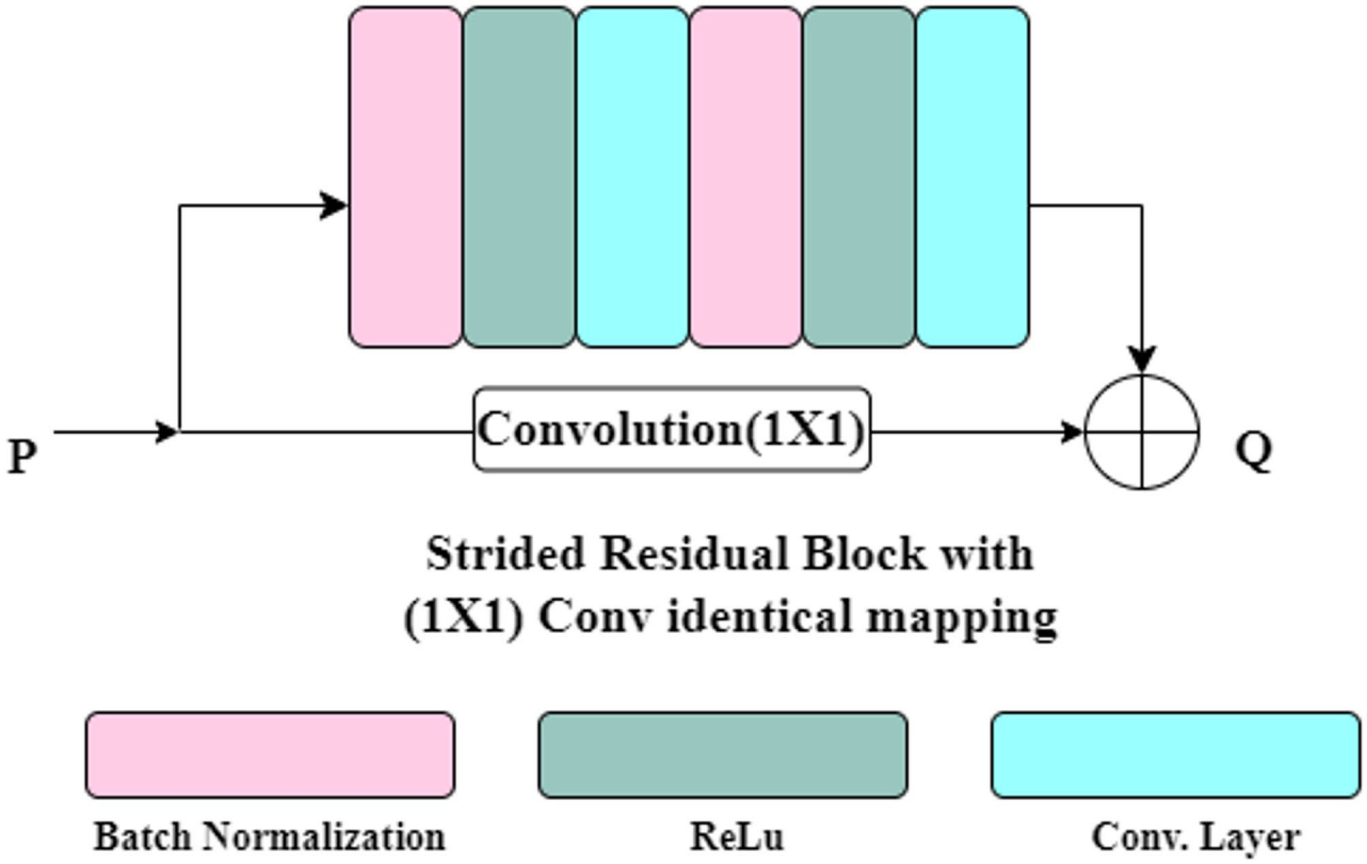
Residual Mapping

To enhance the feature extraction and training stability, the strided residual block incorporates identity residual mapping proposed by He et al.^18^ with batch normalization and ReLU before the convolutional layer in the residual connection. Two sets, each comprising a batch normalization, a ReLU, and a conv2D layer, are stacked in the residual connections. The structure of the strided residual block is represented in Fig. 4. For downsampling, strided convolutional layers with a stride is used instead of pooling layers. In each strided residual block, two conv2D layers have a filter size of 3 × 3, batch normalization layers, and ReLU activation layers. There are 32 filters in the first strided residual block. The second residual block has 64 filters, the third residual block has 128 filters, and the fourth residual block has 256 filters.

**Table 3:** A layer-by-layer comprehensive outline of the maizenet model.

Layer Name	Model Layer	Filter Size	Strides	Output Dimension	Layerwise-Parameters
Input_1	Input	-	-	224 × 224 × 3	0
Cblr_1	Conv2D_BNz_LeakyRelu	3 × 3	1	224 × 224 × 16	432 + 64 + 0
Cblr_2	Conv2D _BNz_LeakyRelu	3 × 3	1	224 × 224 × 32	4640 + 128 + 0
gap_1	GAP-2D	-	-	32	0
reshape	Reshape	-	-	1 × 1 × 32	0
dense	Dense	-	-	1 × 1 × 2	64
dense_1	Dense	-	-	1 × 1 × 32	64
conv2d_1	Conv2D	1 × 1	1	224 × 224 × 1	32
multiply	Multiply	-	-	224 × 224 × 32	0
multiply_1	Multiply	-	-	224 × 224 × 32	0
add_1	Add	-	-	224 × 224 × 32	0
Blrc_1	BNz_LeakyRelu_Conv2D	3 × 3	2	112 × 112 × 32	128 + 0 + 9248
Blrc_2	BNz_LeakyRelu_Conv2D	3 × 3	1	112 × 112 × 32	128 + 0 + 1056
conv2d_2	Conv2D	1 × 1	1	112 × 112 × 32	9248
add_2	Add	-	-	112 × 112 × 32	0
Cblr_3	Conv2D _BNz_LeakyRelu	3 × 3	1	112 × 112 × 64	18,496 + 256 + 0
gap_2	GAP-2D	-	-	64	0
Reshape_1	Reshape	-	-	1 × 1 × 64	0
dense_2	Dense	-	-	1 × 1 × 4	256
dense_3	Dense	-	-	1 × 1 × 64	256
conv2d_3	Conv2D	1 × 1		112 × 112 × 1	64
multiply_2	Multiply	-	1	112 × 112 × 64	0
multiply_3	Multiply	-	1	112 × 112 × 64	0
add_3	Add	-	-	112 × 112 × 64	0
Blrc_3	BNz_LeakyRelu_Conv2D	3 × 3	2	56 × 56 × 64	256 + 0 + 36,928
Blrc_4	BNz_LeakyRelu_Conv2D	3 × 3	1	56 × 56 × 64	256 + 0 + 4160
conv2d_4	Conv2D	1 × 1	1	56 × 56 × 64	36,928
add_4	Add	-	-	56 × 56 × 64	0
Cblr_4	Conv2D _BNz_LeakyRelu	3 × 3	1	56 × 56 × 128	73,856 + 512 + 0
gap_3	GAP-2D	-	-	128	0
reshape	Reshape	-	-	1 × 1 × 128	0
dense_4	Dense	-	-	1 × 1 × 8	1024
dense_5	Dense	-	-	1 × 1 × 128	1024
conv2d_5	Conv2D	1 × 1	1	56 × 56 × 1	128
multiply_4	Multiply	-	-	56 × 56 × 128	0
multiply_5	Multiply	-	-	56 × 56 × 128	0
add_5	Add	-	-	56 × 56 × 128	0
Blrc_5	BNz_LeakyRelu_Conv2D	3 × 3	2	28 × 28 × 128	512 + 0 + 147,584
Blrc_6	BN_LeakyRelu_Conv2D	3 × 3	1	28 × 28 × 128	512 + 0 + 16,512
conv2d_6	Conv2D	1 × 1	1	28 × 28 × 128	147,584
add_6	Add	-	-	28 × 28 × 128	0
Cblr_5	Conv2D _BNz_LeakyRelu	3 × 3	1	28 × 28 × 256	295,168 + 1024 + 0
gap_4	GAP-2D	-	-	256	0
reshape	Reshape	-	-	1 × 1 × 256	0
dense_6	Dense	-	-	1 × 1 × 16	4096
dense_7	Dense	-	-	1 × 1 × 64	4096
conv2d_7	Conv2D	1 × 1	1	28 × 28 × 1	256
multiply_6	Multiply	-	-	28 × 28 × 256	0
multiply_7	Multiply	-	-	28 × 28 × 256	0
add_7	Add	-	-	28 × 28 × 256	0
Blrc_7	BNz_LeakyRelu_Conv2D	3 × 3	2	14 × 14 × 256	1024 + 0 + 590,080
Blrc_8	BNz_LeakyRelu_Conv2D	3 × 3	1	14 × 14 × 256	1024 + 0 + 65,792
conv2d_8	Conv2D	1 × 1	1	14 × 14 × 256	590,080
add_8	Add	-	1	14 × 14 × 256	0
gap_5	GAP-2D	-	-	256	0
dense_8	Dense	-	-	128	32,896
dropout_1	Dropout	-	-	128	0
dense_9	Dense	-	-	64	8256
dropout_2	Dropout	-	-	64	0
dense_10	Dense	-	-	4	260
Total parameters					21,06,388
Trainable parameters					21,03,476
Non-trainable parameters					2,912

### Additional layers

After the fourth residual block, a GAP layer is used. Lin et al.^45^ proposed dual applications of the GAP layer. In one application, GAP substitutes fully connected layers (dense layer) completely, serving as a spatial-wise aggregation to produce a compressed feature vector. In another application, its output is fed to fully connected layers, allowing a combination of spatial information compression and subsequent nonlinear transformations in the network. In this research study, following the GAP, the final FC layers and dropouts are employed to map the extracted features to the final output classes. FC layers are often used in the final stages of the network. These layers link each neuron in one layer to every neuron in the subsequent layer. They correlate extracted features to output classes. An FC layer having 128 neurons and 64 neurons with ReLU activation is used in the proposed model. Neural networks use dropouts as a regularisation method to avoid overfitting during training. It arbitrarily sets a portion of a layer’s neurons to zero to minimize overfitting and drive the network to learn stronger features. In the proposed model, 20% of the neurons in the layer are deactivated during each training step randomly. In the final step, an FC layer with 4 neurons and a SoftMax is used to generate the output, which indicates a classification model for four different classes.


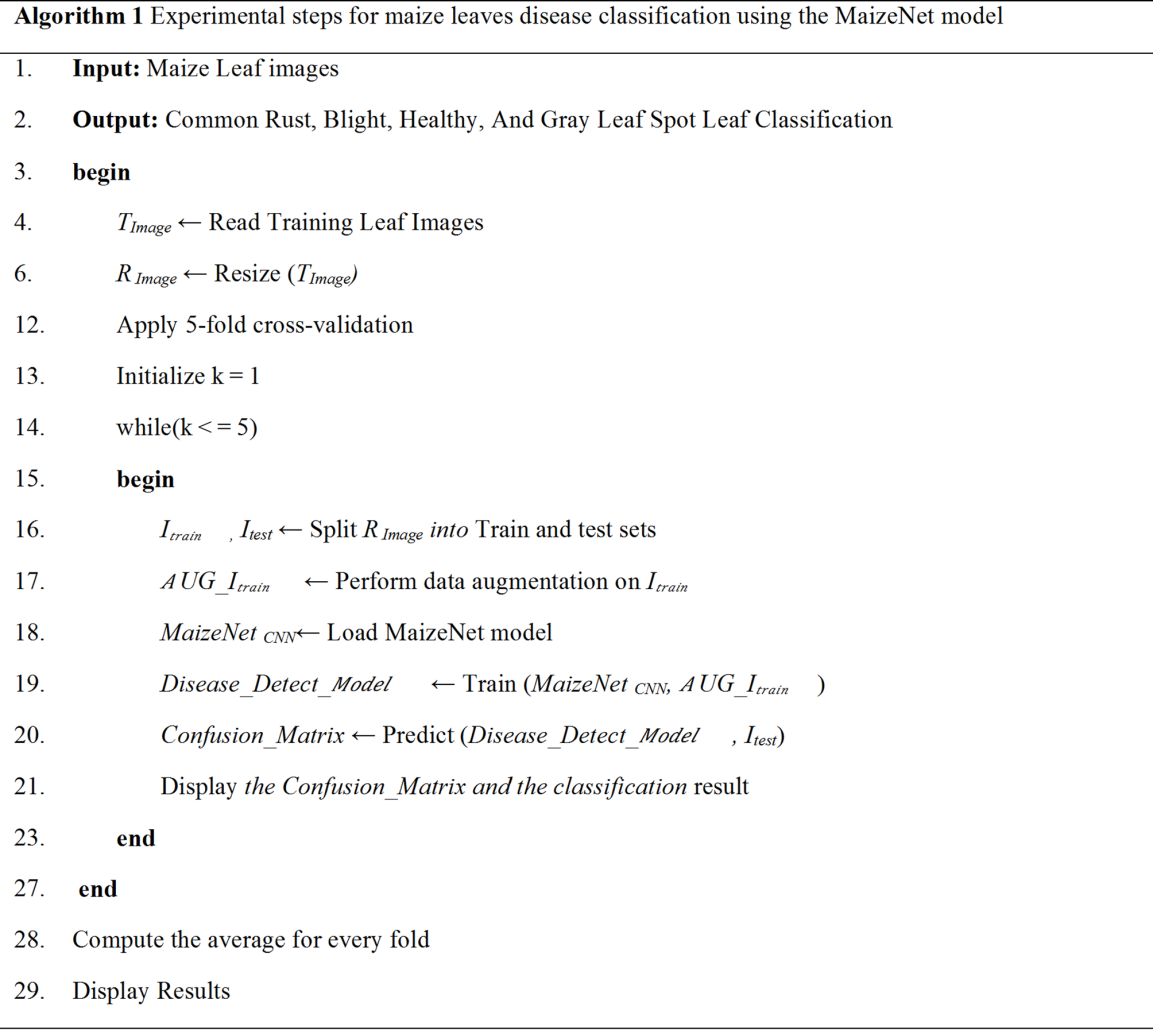


## Experimentation settings and performance evaluation metrics

This section presents the experimentation settings and the performance evaluation metrics used in the study.

### Experimentation details

Google Collaboratory, or Colab, serves as an experimentation hub with 32 GB RAM and an NVIDIA P100 Graphics Processing Unit (GPU) available for 24 h. All the experiments are executed using Python’s Keras library for seamless implementation. For model training, an Adam optimizer and a prescribed learning rate of 0.0005 are used. We have used a dynamic approach for training the model with ReduceLROnPlateau. The combination of rapid adaptation and momentum allows the Adam optimizer to converge fast and with less sensitivity to hyperparameters. Moreover, Table 4 shows the performance of the MaizeNet at different learning rates.

**Table 4:** MaizeNet performance at different learning rate.

Learning rate	Precision	Recall	F1-Score
0.005	0.9308	0.9292	0.9298
0.0005	0.9528	0.9497	0.9509

To improve convergence, a learning rate scheduler was employed with an initial patience of five epochs and a decay factor of 0.5. If there is no performance improvement for five consecutive epochs, it multiplies the learning rate by 0.5, effectively reducing it to half of its current value. Early stopping, employed to mitigate overfitting during model training, incorporates a patience parameter set to 16 epochs. This strategic choice allows the training process to automatically cease if there is no improvement in validation performance for a consecutive 16 epochs, ensuring optimal model complexity and promoting robust generalization. The model is iteratively trained for 120 epochs, each consisting of 16 batch sizes. The average running time/epoch is 31 s for training. The optimizer, number of epochs, patience values are selected based on the prior work. Details of the hyperparameters used in model training are given in Table 5.

**Fig. 5:**
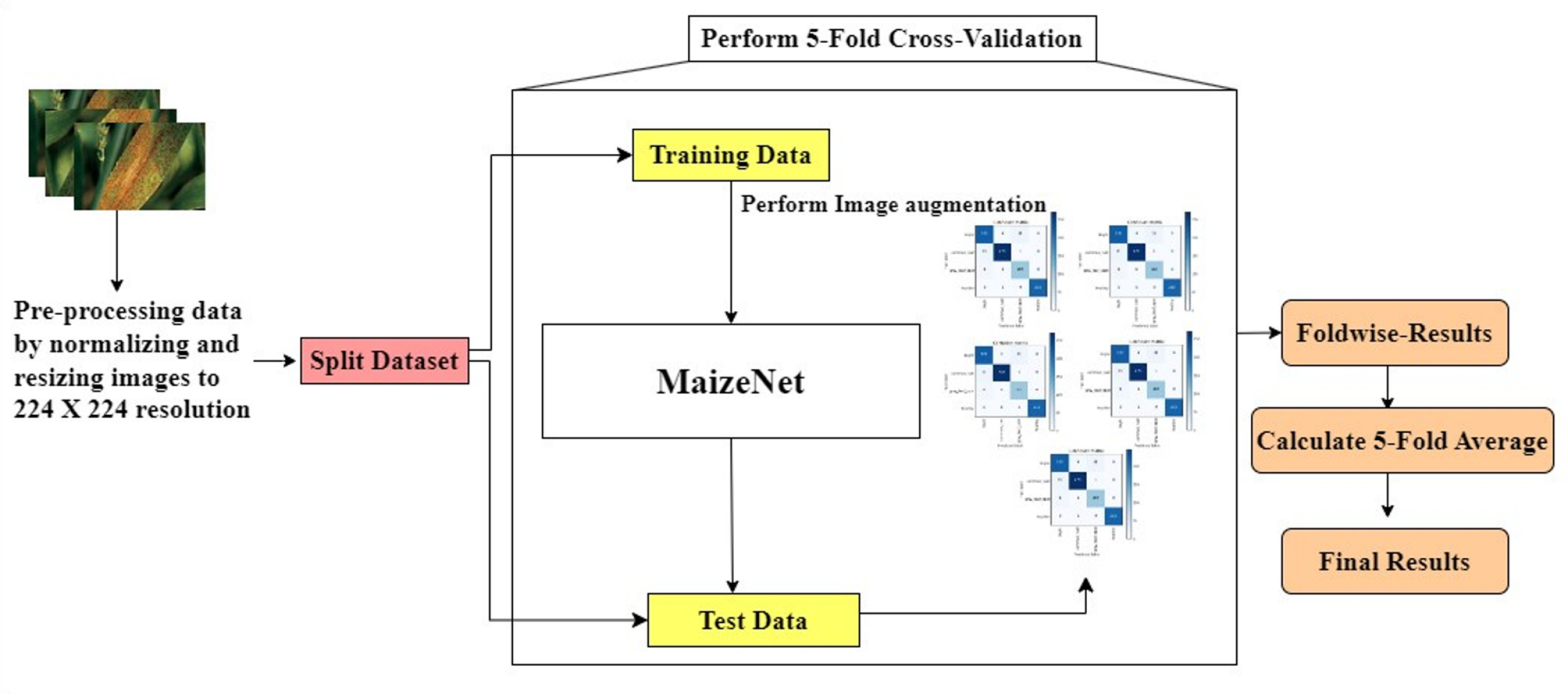
The proposed framework of plant disease classification using the MaizeNet Model

**Fig. 6:**
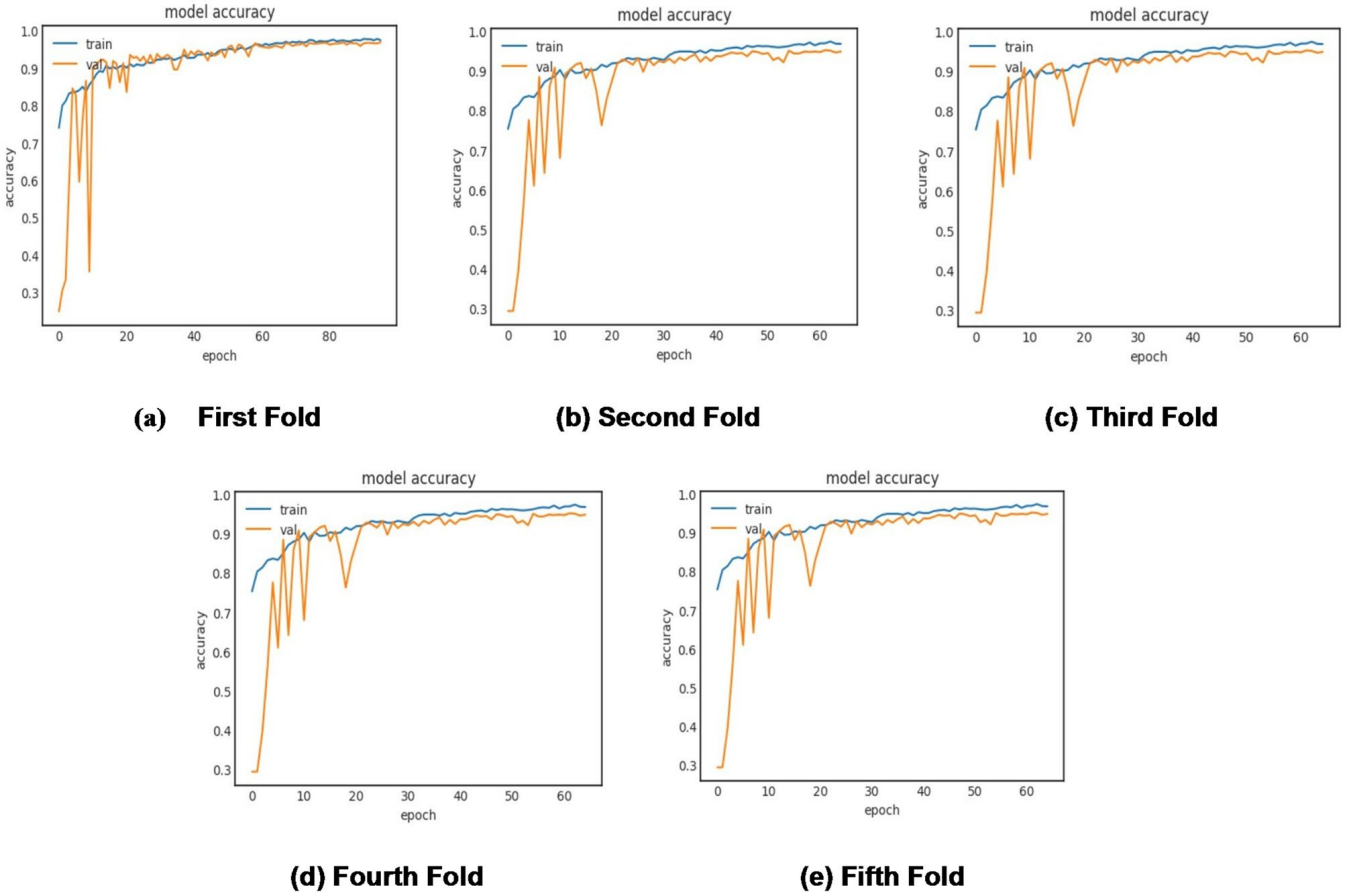
Fold-wise accuracy graphs of the MaizeNet model in training

**Fig. 7:**
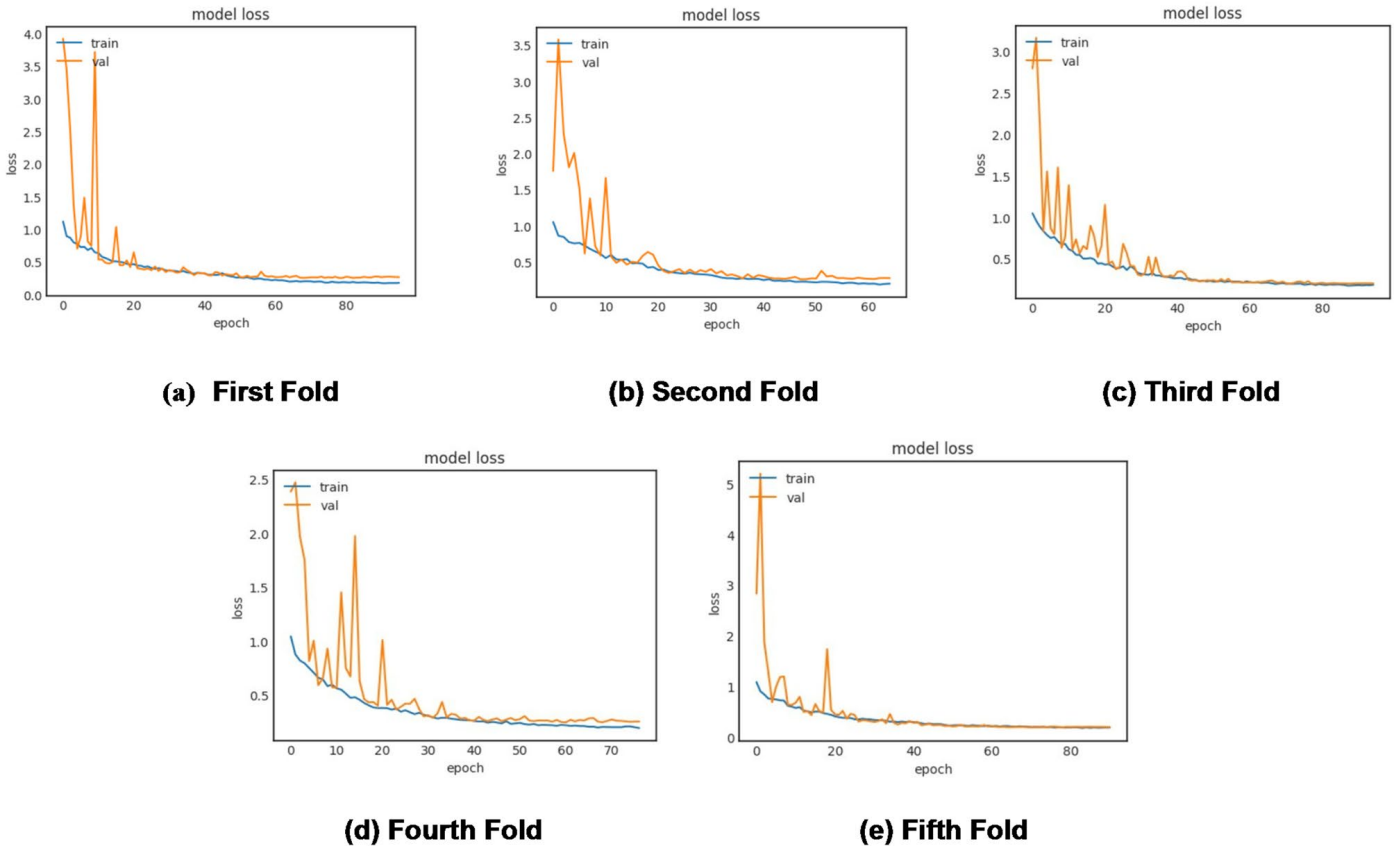
Fold-wise loss of the MaizeNet model in training

The algorithmic steps and proposed framework of plant disease classification using the MaizeNet Model are presented in **Algorithm 1** and Fig. 5. A 5-fold cross-validation approach is employed, with 80% of the input dataset used for model training and 20% representing the test data to evaluate the model’s generalization. This methodology systematically partitions the dataset into five subsets, training the model on four folds while testing on the fifth in each iteration. Five iterations of the method guarantee that every subset acts as a testing set only once. The model’s effectiveness is measured by aggregating performance metrics across all folds, providing a comprehensive assessment of its capability to make generalized decisions on previously unknown data. Figure 6 depicts the fold-wise accuracy graphs of the MaizeNet model during the training phase. Figure 7 illustrates the loss graphs depicting the convergence of training and validation loss across all 5-folds for the MaizeNet model. The absence of divergence indicates that the model avoids overfitting, demonstrating its robustness in capturing underlying patterns in the data.

**Table 5:** Details of hyperparameters used in model training.

Hyper-parameter	Values
Optimizer	Adam
Loss Function	Categorical Cross-Entropy
Epochs	120
Batch-Size	16
Early Stopping (Yes/No)	Yes
Learning Rate	0.0005
Patience	16

Moreover, this study employs categorical cross-entropy as the loss function, quantifying dissimilarity between true and predicted class distributions by calculating the negative log-likelihood across multiple classes. The categorical cross-entropy loss function is mathematically defined using the following **formula below**:

2 $$\:L\left(y,\widehat{y}\right)=-{\sum\:}_{i}{y}_{i}\bullet\:{log}(\widehat{{y}_{i}})$$

Here $\:y$ represents the true distribution and $\:\widehat{y}\:$is the predicted distribution, with $\:i$ iterating over the categories.

### Performance evaluation metrics

In this study, accuracy, precision, recall, and F1-score are employed to evaluate the maize disease classification algorithm. Precision improves the model’s ability to identify diseased plants accurately while reducing the number of false positives. Recall assesses how effectively it can identify every instance of a specific class. F1-score, being the harmonic mean of recall and precision, gives a fair evaluation of the effectiveness of a model, while the overall validity of the predictions is represented by accuracy. Confusion metrics enhance the completeness of performance analysis by offering a concise overview of a model’s recall, F1-score, accuracy, and precision, enabling a more in-depth analysis of its benefits as well as its drawbacks in the context of class differentiation.

The mathematical formulas for F1-score, precision(P), recall (R or Sensitivity), and accuracy are **listed below**:

3 $$P = \frac{{TP}}{{TP + FP}}$$

4 $$R = \frac{{TP}}{{TP + FN}}$$

5 $$F1\;score = 2 \times \frac{{P \times R}}{{P + R}}$$

6 $$Accuracy = \frac{{TP + TN}}{{TP + TN + FN + FP}}$$

Here, the numbers for true positives (TP), true negatives (TN), false positives (FP), and false negatives (FN) are indicated. These metrics are commonly used in binary and multiclass classification tasks to evaluate the performance of a model.

**Table 6:** Five-fold cross-validated results for blight-diseased leaves.

Class	Fold	Precision	Recall	F1 score
Blight	1	0.9349	0.9526	0.9437
-	2	0.9127	0.9167	0.9147
-	3	0.9035	0.9669	0.9341
-	4	0.9212	0.9367	0.9289
-	5	0.9348	0.9430	0.9389
Average	-	0.9214	0.9431	0.9320

**Table 7:** Five-fold cross-validated results for common rust diseased leaves.

Class	Fold	Precision	Recall	F1 score
Common Rust	1	0.9882	0.9805	0.9844
-	2	0.9462	0.9762	0.9609
-	3	0.9919	0.9762	0.9840
-	4	0.9854	0.9574	0.9712
-	5	0.9925	0.9634	0.9777
Average	-	0.9808	0.9707	0.9756

**Table 8:** Five-fold cross-validated results for Gray leaf spot diseased leaves.

Class	Fold	Precision	Recall	F1 score
Gray Leaf Spot	1	0.9231	0.9000	0.9114
-	2	0.9083	0.8462	0.8761
-	3	0.9314	0.8190	0.8716
-	4	0.8947	0.9273	0.9107
-	5	0.9043	0.9369	0.9204
Average	-	0.9123	0.8858	0.8980

**Table 9:** Five-fold cross-validated results for healthy leaves.

Class	Fold	Precision	Recall	F1 score
Healthy	1	0.9961	1.0000	0.9981
-	2	1.0000	0.9960	0.9980
-	3	0.9958	1.0000	0.9979
-	4	1.0000	1.0000	1.0000
-	5	0.9915	1.0000	0.9957
Average	-	0.9966	0.9992	0.9979

**Table 10:** Macro average among blight, common rust, Gray leaf spot and healthy leaf images.

Fold	Precision	Recall	F1 score
1	0.9606	0.9583	0.9594
2	0.9418	0.9337	0.9374
3	0.9556	0.9405	0.9469
4	0.9503	0.9554	0.9527
5	0.9558	0.9608	0.9582
Average	0.9528	0.9497	0.9509

**Table 11:** Weighted average among blight, common rust, Gray leaf spot and healthy leaf images.

Fold	Precision	Recall	F1 score
1	0.9681	0.9681	0.9680
2	0.9477	0.9480	0.9476
3	0.9594	0.9586	0.9582
4	0.9593	0.9586	0.9588
5	0.9651	0.9645	0.9647
Average	0.9599	0.9595	0.9594

## Results and discussion

**Fig. 8:**
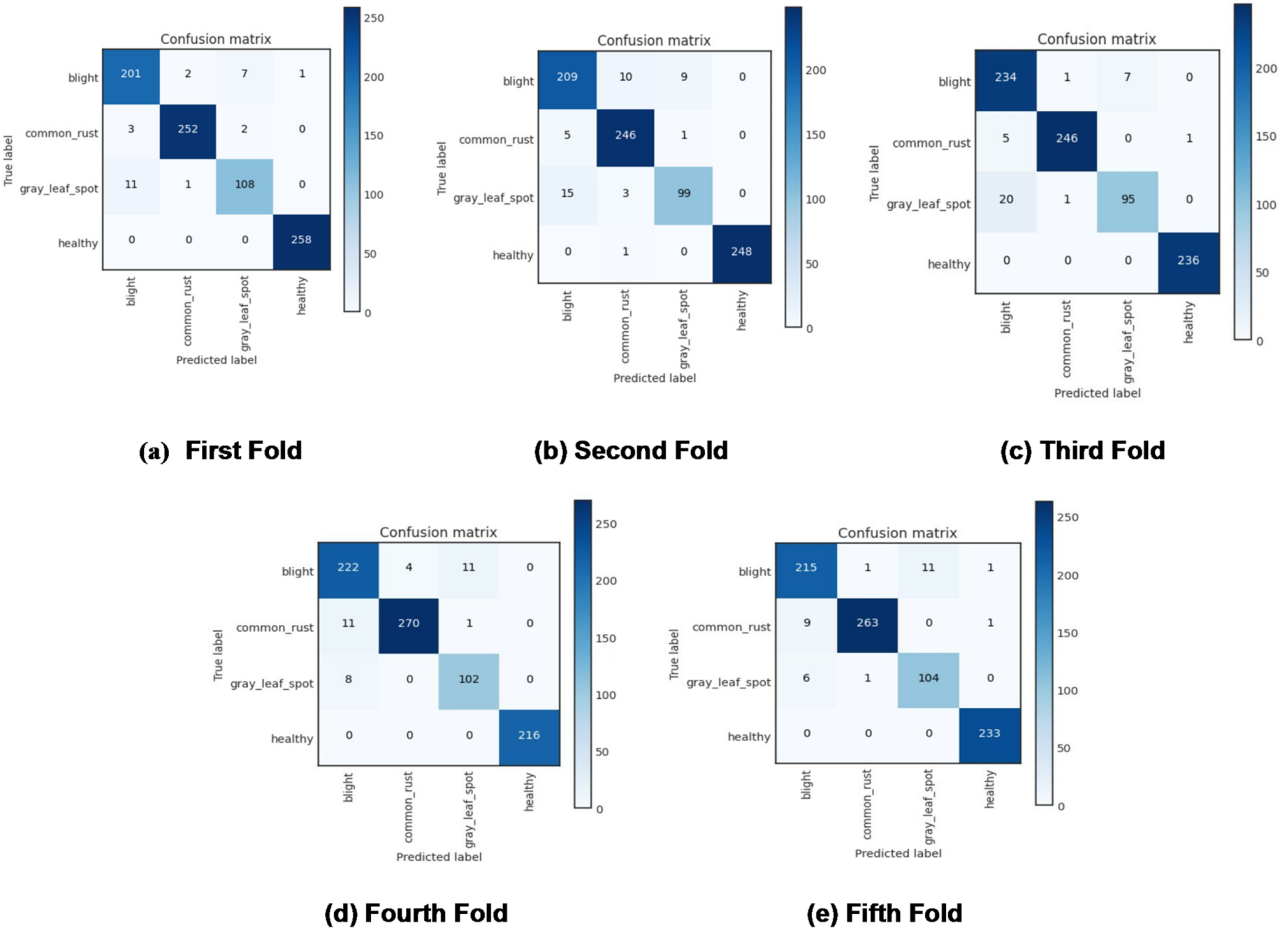
Fold-wise confusion matrix of MaizeNet model in training

The current section covers the experimental observations of the MaizeNet model. Moreover, the MaizeNet performance in comparison to that of other maize disease classification architectures is presented in this section. Figure 8 demonstrates the confusion metrics at each fold for each leaf class. Tables 6, 7, 8 and 9 show the performance results for each fold of blight, common rust, gray leaf spot, and healthy class. Figure 8 also shows that the proposed MaizeNet model incorrectly classified some instances between the four-leaf classes.

For the blight class, poorer performance figures are given in fold 2. The lowest precision of 0.9127, the lowest recall of 0.9167, and the lowest F1-score value of 0.9147 are attained in this fold. Fold 1 exhibits higher performance values. In fold 1, a Precision value of 0.9349, a recall value of 0.9526, and an F1-score value of 0.9437 are obtained. For the blight class, the average value of precision in fold 1 is 0.9214, the average recall of 0.9431, and the average F1-score value is 0.9320. In the case of common rust, the lower values of precision and F1 score of 0.9462 and 0.9609 are stated in fold 2, while the lower recall value is reported in fold 4. Fold 5 provides a higher precision value of 0.9925. A higher F1-score value of 0.9844 and a recall value of 0.9805 are achieved in fold 1. For of common rust class, the average precision of 0.9808, recall value of 0.9707, and F1-score value of 0.9756 are obtained across all folds.

For gray leaf spot, the lowest precision of 0.8947 is reported in fold 4, while fold 3 reports the lowest recall value of 0.8190 and the lowest F1-score of 0.8716. Fold 3 shows the highest precision value of 0.9314 while fold 5 reports the highest recall, and the highest F1-score values as 0.9369 and 0.9204. In the gray leaf spot class, the average values of precision, recall, and F1-score are 0.9123, 0.8858, and 0.8980, respectively. The model performs better with higher precision value, recall, and F1-score in the case of the healthy leaf class. The lowest precision value of 0.9915 and the lowest F1-score achieved is 0.9957, are obtained in fold 5, while fold 2 reports the lowest recall value of 0.9960. Folds 2 and 4 achieve the highest precision value of 1.0000, while the highest recall value of 1.0000 is obtained in all folds except fold 2. Fold 4 achieves the highest F1-score value of 1.0000. In fold 4, all the metrics are reported as 1.0000. The average values obtained for precision, recall, and F1-score in the case healthy class are 0.9966, 0.9992, and 0.9979, respectively. Furthermore, the highest number of misclassifications is reported in the gray leaf spot class, while the best classification results are achieved in the healthy leaf class.

The macro average in the 5-fold validation minimizes the implications of class imbalance and provides balanced evaluation across all the classes. It ensures that each class has equal weightage, checks for variations in performance, and strengthens the model’s robustness to a broad spectrum of data sets. The macro average facilitates the interpretation and validation of the model’s cross-fold generalization by offering a complete point of view. In the context of 5-fold cross-validation, the macro average for a particular metric is usually determined by calculating the average performance over all folds for each class and then computing the mean of the class averages. Let us consider that$\:\:{m}_{i,j}$ denotes any evaluation metric for a specific class in a fold $\:i$ as the fold index ranges from 1 to 5 and $\:j$ it is the class index. The following formula computes the macro average (MA):

7 $$MA = \frac{1}{N}\sum\limits_{j = 1}^N {\frac{1}{5}} \sum\limits_{i = 1}^5 {{m_{i,j}}}$$

Here, the number of classes is indicated by N. Since there are a total of four-leaf classes in the present research, N obtains a value of 4. In Table 10, the macro averages of all evaluation metrics are reported. The macro-averaged precision value, recall value, F1-score, and accuracy of MaizeNet are reported as 0.9528, 0.9497, 0.9509, and 0.9595, respectively. We also used the weighted average, considering class sizes, to show the practical effectiveness of the model’s predictions across all classes. We attempted to present the model’s performance by employing both metrics, considering both the equal treatment of classes and the real-world implications of classification on the unbalanced dataset. The formula for calculating the weighted average (WA) for N classes is as follows:

8 $$WA = \sum\limits_{j = 1}^N {{w_j}} \times {m_j}$$

Here $\:{w}_{j}\:$is the weight given to the class $\:j$ based on its prevalence and $\:{m}_{j}$ is the value of the evaluation metric for the class $\:j$. The weighted average precision value, recall value, F1-score, and accuracy of the MaizeNet model are reported as 0.9599, 0.9595, 0.9594, and 0.9595, respectively, as shown in Table 11. Since the model has performed well, there is no significant difference between the macro-average and weighted average.

Macro F1 score provides a more balanced and reliable measure of the model’s effectiveness in handling all four maize disease categories, including underrepresented ones. This choice is particularly suitable for imbalanced multiclass classification tasks, as it directly reflects the model’s ability to generalize across all classes, not just the dominant ones. The F1-score achieved by MaizeNet shows the generalization of the model.

MaizeNet performance is compared with nine existing models developed for maize crop disease classification. Table 12 shows the model’s comparative results with previous works. The one-on-one comparison is not feasible because of variations in technology, number of samples, simulation settings, parameter settings, and methodology, which limit the scope of this comparison. The dataset that is used to train and test the proposed MaizeNet contains images of maize leaves captured in lab settings and the images acquired in actual field settings with complex backgrounds and inconsistent lighting strengths, like diverse field scenarios, changing soil colours, bright and sunny, or cloudy weather scenarios.

To prevent biases and evaluate the effectiveness of our method thoroughly, a five-fold cross-validation test is performed. This generated five models built on five distinct training sets based on the partitioned datasets. According to Table 11, the recall ranges from 0.9480 to 0.9681, confirming that dataset partitioning does not influence the model’s performance. Further, five-fold cross-validation has an average classification accuracy of 0.9595. The classification results support the hypothesis that the proposed CNN could identify and categorize maize leaf diseases.

For the maize sample taken from the Plant Village dataset, the models developed by Chen et al.^22^ and Sibiya et al.^23^ were not precise enough to recognize the diseased area with prediction accuracies of 0.8425 and 0.8900. In comparison, the model produced by Sibiya et al.^33^ has more effective results on maize images from Plant Village datasets with an accuracy of 0.9285. The model proposed by Xu et al.^27^ performed with comparable 0.9600 classification accuracy with precision, recall, and F1-score of 0.9600 on the plant village dataset, but on another dataset comprising images captured in real backgrounds, the model’s accuracy was reduced to 0.9328.

**Table 12:** Comparison of the proposed technique to those identified in the literature for the maize crop.

Papers	Model	Dataset	Class	Source	Training Strategy	Accuracy	F1-Score	Recall	Precision
[33]	CNN	Own dataset	4	Lab Conditions	70:30 train-test division	0.9285	-	-	-
[22]	VGG-Net	Plant Village	4	Lab Conditions	70:30 train-test division	0.8425	-	0.6850	-
		Own dataset	4	Field conditions	70:30 train-test division	0.8038	-	0.6076	-
[44]	VGG-16	Plant Village	4	Lab Conditions	80:10:10 train-validation-test division	0.8900	-	-	-
[17]	Mobile-DANet	Plant Village	4	Lab Conditions	80:20 train-test division	0.9850	0.9700	0.9900	0.9700
[25]	Inception-v3	Own dataset	4	Field conditions (brightened)	70:15:15 train-validation-test division	0.9599	0.9594	0.9596	0.9594
[29]	SSD + TM + RPN + GloU512	NLB dataset	2	Field conditions	50:40:10 train-validation-test division	-	-	-	0.9183
[27]	Modified AlexNet	Plant Village	4	Lab Conditions	80:20 train-test division	0.9600	0.9600	0.9600	0.9600
		Own Dataset	4	Field conditions	80:20 train-test division	0.9328	-	-	-
[37]	CNN + ViT	Dataset used by [22]	4	Field conditions	70:30 train validation division	0.9259	0.9193	0.9160	0.9250
[45]	VGG-Inception	Dataset used by [22]	4	Field conditions	70:30 train validation split	0.9136	-	-	-
[46]	ResNet-50	PlantVillage + Plant Doc	4	Lab Conditions + Field Conditions	10-fold Cross Validation	0.9400	0.9300	0.9200	9.9500
[46]	ResNet-34	PlantVillage + Plant Doc	4	Lab Conditions + Field Conditions	10-fold Cross Validation	0.9300	0.9200	0.9100	0.9400
This study		Plant Village + Plant Doc	4	Lab Conditions + Field Conditions	5-Fold Cross-Validation	0.9595	0.9594	0.9595	0.9599

When training the models on the images collected in field conditions, the attention-based CNN proposed by Chen et al.^22^ also achieved a not-so-good accuracy of 0.8028 and a precision value of 0.7600. Thakur et al.^37,46^ built their models by training the dataset used by Chen et al.^22^. Thakur et al.^37^ achieved an accuracy of 0.9259, a recall of 0.9160, a precision of 0.9250, and an F1-score of 0.9193. In the recent work of the same researchers, Thakur et al.^46^, the model comprised significantly reduced parameters (6 million). With a limited storage requirement, it is ideal for applications on smartphones and tablets, with a compromise of attaining a classification accuracy of 0.9136 only. In another recent research study on maize leaf disease classification by Haque et al.^25^, the inception v3-based model was performed with approximately similar classification results as the proposed MaizeNet model. Their model achieved 0.9571 accuracy in classifying images captured in normal conditions, while 0.9599 accuracy was achieved in brightness-enhanced images. Sun et al.^29^ worked on the NLB dataset, and their feature fusion-based model classified the maize images with a precision of 0.9183. Theerthagiri et al.^38^ evaluated the pre-trained VGG-16, ResNet-34, ResNet-50 models for the maize disease classification on PlantVillage dataset. The VGG-16 model obtained the accuracy of 0.92, recall of 0.90, and F1-score of 0.91. While ResNet-50 achieved the accuracy of 0.94, recall of 0.92, and precision of 0.95.

The results demonstrate that, compared to some of the present models, the MaizeNet model is more accurate at classifying diseases. The model introduced by Chen et al.^16^ produced superior classification results. Although their model used a plant village dataset comprised of images acquired under laboratory settings. Using images taken by low-cost mobile phones used by farmers is crucial to adapt to natural conditions with significant noise in the background. Dataset used in this study includes images that are captured in the field without controlling background or light settings. Nevertheless, rather than using the cross-validation approach that we performed to evaluate MaizeNet, the performance of other models was assessed by employing only one train-test split. Again, the cross-validation performance assessment is encouraged since it produces more generic results. The experiments showed satisfactory outcomes, but there are some dataset constraints to overcome in future studies. A more diversified dataset comprising images of plant leaves acquired under varying weather conditions with natural backgrounds is required. The proposed model requires extensive data to validate its classification performance.

Moreover, the Kruskal-Wallis test, a non-parametric statistical test is applied for comparing distributions across classes. Specifically, the test shows the observed differences in precision across classes were statistically significant. The Kruskal-Wallis test yielded p-value of 0.0090 **(***p <* 0.01), indicating a statistically significant difference in performance between classes.

**Table 13:** Results of the ablation studies.

Ablation Study	F1-Score	Recall	Precision
without strided residual block	0.9523	0.9517	0.9535
without csSE attention mechanism	0.9433	0.9388	0.9497
MaizeNet model	0.9594	0.9595	0.9599

### Ablation study

Table 13 shows the results of the two ablation studies. These studies are conducted to investigate the contribution of strided residual learning and csSE attention mechanism in the performance of the model. In the first study, csSE attention layer is removed to study the impact of attention mechanism in the MaizeNet model. When the model was evaluated, the precision, F1 score, and recall dropped as shown in Table 13. In the second study, the strided residual block is replaced with the convolutional block and pooling layer. When the modified model was evaluated the precision, recall, and F1 score dropped in comparison to the MaizeNet model. This shows the strided residual layer helps in capturing better feature extraction. So, the ablation study shows that the attention mechanism and the strided residual layer helps in useful feature extraction that increased the performance of the MaizeNet model for maize disease classification.

### Limitations

Only 350 of the images were taken in real fields, while the rest came from controlled lab environments. So, the model may not fully capture how leaves look in different natural conditions. Since real-field conditions are more complex, the model may not perform as well outside lab-like scenarios. The model performed slightly lower on gray leaf spot compared to other classes, possibly due to fewer or more varied samples. Despite this, the model showed high accuracy, suggesting the method is effective and can be improved further with more diverse field data.

Moreover, for better understanding the limitations of the study, a subset of misclassified samples is also analysed. Most errors occurred between gray leaf spot and blight, likely due to overlapping visual characteristics such as irregular brown lesions. Additionally, a small number of healthy leaves were misclassified as diseased, particularly when leaves showed signs of physical damage or shadowing that resembled disease symptoms. Notably, some real-field images were misclassified more frequently than lab-controlled ones, indicating that background clutter, lighting variations, and image noise impact the model’s accuracy. These observations suggest that enhancing field data diversity and incorporating robust pre-processing or augmentation methods could improve real-world performance.

### Future work

To further enhance the robustness and practical applicability, the proposed model will have to trained and tested on plant images captured in natural agricultural environments. Extending the model to support more plant species and a wider range of diseases will make it more useful for large-scale agricultural applications. Future directions may also involve optimizing the model for deployment on mobile or edge devices to enable real-time disease detection in the field.

## Conclusion

The present study presents a deep CNN model named MaizeNet. The MaizeNet model uses innovative techniques such as residual learning and a channel spatial squeeze-excitation network to improve the extraction of features, along with training stability. The dataset that includes images taken in both lab and natural environments verifies the effectiveness of the proposed approach. Evaluation measures such as micro-averaged and weighted F1-score, recall, precision, and accuracy show that the model can correctly classify plant leaves. The observations of the 5-fold cross-validation test show the stability of MaizeNet model. It reveals that data partitioning has no adverse impact on the model’s predictive performance. With an average classification accuracy of 0.9595, MaizeNet is an effective method for precisely and automatically identifying plant diseases in maize leaves. These outcomes demonstrate MaizeNet robustness and reliability in automated plant disease classification.

## Data Availability

The dataset is publicly available at https://www.kaggle.com/datasets/smaranjitghose/corn-or-maize-leaf-disease-dataset.
